# Blood coagulation in Prediabetes clusters–impact on all-cause mortality in individuals undergoing coronary angiography

**DOI:** 10.1186/s12933-024-02402-z

**Published:** 2024-08-22

**Authors:** Sebastian Hörber, Katsiaryna Prystupa, Johann Jacoby, Andreas Fritsche, Marcus E. Kleber, Angela P. Moissl, Peter Hellstern, Andreas Peter, Winfried März, Robert Wagner, Martin Heni

**Affiliations:** 1 Institute for Clinical Chemistry and Pathobiochemistry, Department for Diagnostic Laboratory Medicine, University Hospital Tübingen, Tübingen, Germany; 2 Institute of Diabetes Research and Metabolic Diseases, Helmholtz Center Munich German Research Center for Environmental Health, Tübingen, Germany; 3 German Center for Diabetes Research, Neuherberg, Germany; 4 Institute for Clinical Diabetology, German Diabetes Center (DDZ), Leibniz Center for Diabetes Research at Heinrich-Heine University, Düsseldorf, Germany; 5 Institute for Clinical Epidemiology and Applied Biometry, University Hospital Tübingen, Tübingen, Germany; 6 Department for Diabetology, Endocrinology, and Nephrology, University Hospital Tübingen, Tübingen, Germany; 7 Vth Department of Medicine (Nephrology, Hypertensiology, Rheumatology, Endocrinology, Diabetology), Medical Faculty Mannheim, University of Heidelberg, Mannheim, Germany; 8 SYNLAB MVZ für Humangenetik Mannheim GmbH, Mannheim, Germany; 9 Center of Hemostasis and Thrombosis Zurich, Zurich, Switzerland; 10 SYNLAB Academy, SYNLAB Holding Deutschland GmbH, Augsburg and Mannheim, Munich, Germany; 11 Clinical Institute of Medical and Chemical Laboratory Diagnostics, Medical University of Graz, Graz, Austria; 12 Division of Endocrinology and Diabetology, Department of Internal Medicine 1, University Hospital Ulm, Ulm, Germany

**Keywords:** Prediabetes, Cluster, Coagulation, Mortality

## Abstract

**Background:**

Metabolic clusters can stratify subgroups of individuals at risk for type 2 diabetes mellitus and related complications. Since obesity and insulin resistance are closely linked to alterations in hemostasis, we investigated the association between plasmatic coagulation and metabolic clusters including the impact on survival.

**Methods:**

Utilizing data from the Ludwigshafen Risk and Cardiovascular Health (LURIC) study, we assigned 917 participants without diabetes to prediabetes clusters, using oGTT-derived glucose and insulin, high-density lipoprotein cholesterol, triglycerides, and anthropometric data. We performed a comprehensive analysis of plasmatic coagulation parameters and analyzed their associations with mortality using proportional hazards models. Mediation analysis was performed to assess the effect of coagulation factors on all-cause mortality in prediabetes clusters.

**Results:**

Prediabetes clusters were assigned using published tools, and grouped into low-risk (clusters 1,2,4; *n* = 643) and high-risk (clusters 3,5,6; *n* = 274) clusters. Individuals in the high-risk clusters had a significantly increased risk of death (HR = 1.30; CI: 1.01 to 1.67) and showed significantly elevated levels of procoagulant factors (fibrinogen, FVII/VIII/IX), D-dimers, von-Willebrand factor, and PAI-1, compared to individuals in the low-risk clusters. In proportional hazards models adjusted for relevant confounders, elevated levels of fibrinogen, D-dimers, FVIII, and vWF were found to be associated with an increased risk of death. Multiple mediation analysis indicated that vWF significantly mediates the cluster-specific risk of death.

**Conclusions:**

High-risk prediabetes clusters are associated with prothrombotic changes in the coagulation system that likely contribute to the increased mortality in those individuals at cardiometabolic risk. The hypercoagulable state observed in the high-risk clusters indicates an increased risk for cardiovascular and thrombotic diseases that should be considered in future risk stratification and therapeutic strategies.

**Graphical Abstract:**

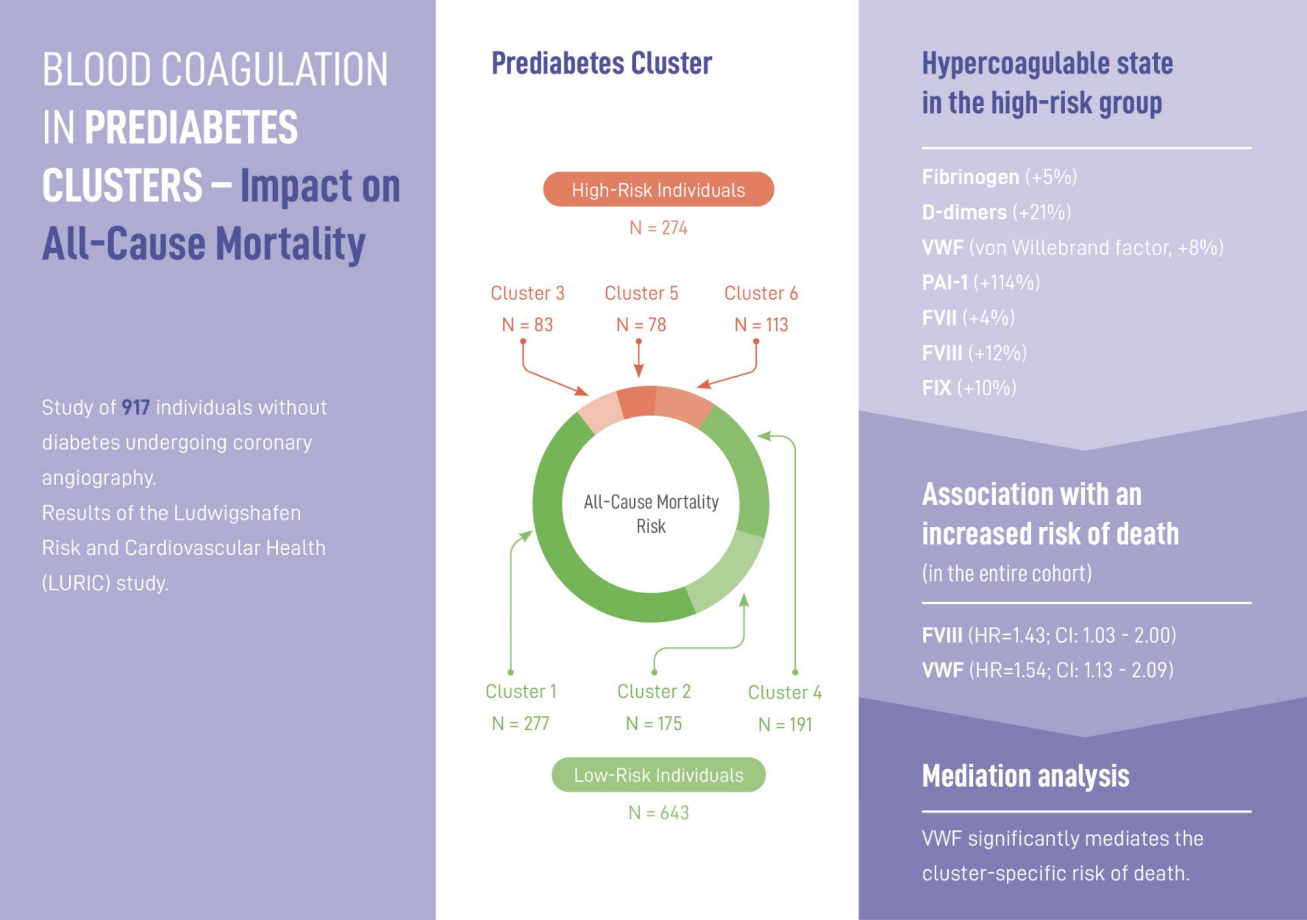

**Supplementary Information:**

The online version contains supplementary material available at 10.1186/s12933-024-02402-z.

## Introduction

Prediabetes is a heterogeneous condition that predisposes those affected not only to a progression to diabetes mellitus, and predicts further complications, but is even linked to increased mortality risk [1–4]. Recently, we identified six pathophysiologic subphenotypes of prediabetes that stratify individuals without diabetes into distinct metabolic clusters. The clustering approach was based on attributes of glucose and lipid-metabolism, body fat distribution and liver fat content as well as genetic risk factors [5]. The resulting subgroups had a markedly different risk for the progression towards type 2 diabetes, vascular complications, and mortality. Patients in two of the identified subgroups (clusters 3 and 5) experience the highest risk of diabetes progression among all clusters and have a high cardiovascular risk. Individuals in cluster 6 have elevated glycemia at baseline, but have a lower risk of diabetes progression compared to clusters 3 and 5. Instead, they have a higher risk of kidney disease and all-cause mortality, despite their relatively low risk for type 2 diabetes. The remaining subgroups (clusters 1, 2 and 4) are characterized by a low risk of diabetes progression and a lower risk of related complications and all-cause mortality. Recently, this clustering has been replicated in individuals with clinical suspicion of cardiovascular disease [6]. Here, the previously defined clusters predict differences in survival and can identify individuals at risk for premature death.

Focusing on the association between prediabetes and cardiometabolic complications, meta-analyses have shown that, compared with normoglycemia, prediabetes is associated with an increased risk of atherosclerotic cardiovascular disease and all-cause mortality [1]. This might be, at least in parts, due to links between prediabetes and the coagulation system as studies demonstrated a strong link between metabolic abnormalities in prediabetes and alterations in the coagulation system [7–10]. For example, obesity and insulin resistance are associated with an increased risk of arterial and venous thrombosis and subsequent cardiovascular disease, resulting from a prothrombotic and proinflammatory milieu [9, 11, 12]. Hyperglycemia and insulin resistance have been found to be associated with a prothrombotic state independent of body weight [8, 13]. In detail, elevated blood glucose concentrations are associated with elevated levels of prothrombotic coagulation factors such as FVIII and FIX. Furthermore, studies have shown that individuals with prediabetes have elevated levels of von Willebrand factor (vWF) and plasminogen activator inhibitor-1 (PAI-1), both markers of endothelial dysfunction [14, 15]. In addition, the plasmatic coagulation system is involved in the progression of atherosclerotic plaques, thereby increasing the risk of atherosclerotic vascular disease (ASCVD), including coronary artery disease, peripheral artery disease, and ischemic stroke [16]. Some studies have examined whether changes in coagulation factors are associated with increased all-cause mortality [17–20]. However, these data are often conflicting and often focus on single parameters of the coagulation system and do not specifically address differences in prediabetes or diabetes mellitus clusters.

Given the major differences in cardiovascular disease between the newly defined prediabetes clusters, we hypothesize that alterations of the coagulation system exist and could contribute to the cluster-specific risk of all-cause mortality. As this relationship may have implications for risk stratification and clinical management of individuals with prediabetes and has not been previously studied, it represents an unresolved open research question that requires further investigation. We therefore examined the association between prediabetes clusters and the coagulation system and addressed the potential impact on survival.

## Materials and methods

### Study background and participants

The Ludwigshafen Risk and Cardiovascular Health (LURIC) Study is a prospective study of individuals who underwent coronary angiography (for details see reference [21]). The aim of the study has been to characterize individuals at risk for cardiovascular disease and to identify prognostic biomarkers for prevention strategies. Upon enrollment, all participants completed a standardized questionnaire and underwent comprehensive laboratory testing. Information on participant deaths and causes of death were obtained from local registries.

**Fig. 1:**
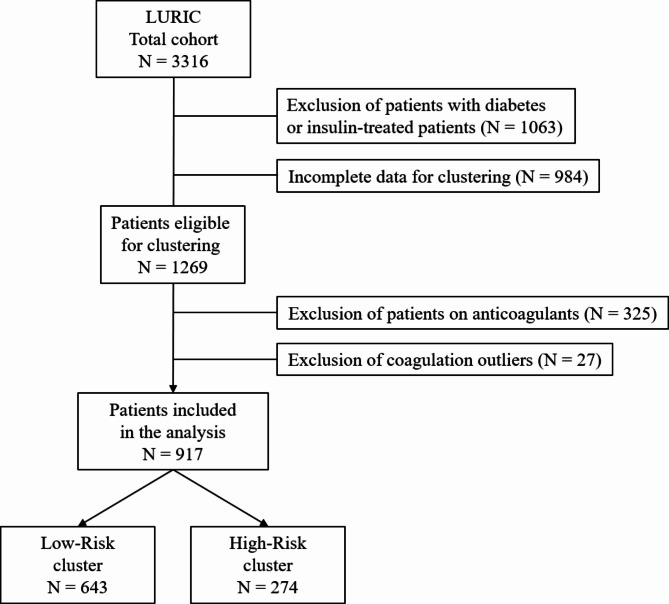
Flow chart of patient selection for the present analysis

A total of 3316 individuals were enrolled. Our analysis was restricted to participants without diabetes and for whom the variables needed for clustering were available (see Fig. 1). This resulted in a subset of 1269 eligible participants. Because coagulation assays are influenced by anticoagulant drugs, participants receiving vitamin K antagonists or heparins were excluded (*n* = 325). In addition, individuals with outliers of coagulation parameters were excluded (*n* = 27, see Supplementary Table S1). Finally, a total of 917 participants was considered in the analysis.

### Prediabetes clustering

Stratification of the analyzed cohort was performed according to the previously published prediabetes clustering approach [5, 6]. In detail, an online application (http://www.bit.ly/PrediabetesCluster) was used to build six clusters based on glucose and insulin measurements before and after 120 min of a 75 g glucose tolerance test (oGTT), fasting triglycerides, high-density lipoprotein cholesterol, BMI, and waist and hip circumference.

### Coagulation measurements

Blood samples were collected as described elsewhere [21]. Coagulation measurements were performed immediately after blood collection in citrate-containing plasma on a STA Stago (Stago Diagnostica, Roche Mannheim, Germany) using the following reagents: aPTT: STA APTT Kaolin; coagulation factors II and VII: PT-based one stage clotting assay, FII/VII deficient plasma (Immuno GmbH, Heidelberg, Germany); coagulation factors VIII, IX, XI: aPTT-based one stage clotting assay, FVIII/IX/XI deficient plasma (Immuno GmbH); D-dimers STA LIATEST D-Di (Stago Diagnostica); fibrinogen: Clauss method, STA fibrinogen (Stago Diagnostica); prothrombin time: STA Neoplastin Plus^®^ (Stago Diagnostica); von Willebrand factor (vWF) antigen: STA Liatest^®^ vWF (Stago Diagnostica). Plasmin-activator inhibitor-1 activity (PAI-1) was measured on a SLT Spectra TECAN with the Chromolize™ PAI-1 test (Biopool, Umea, Sweden). All coagulation measurements were performed using commercially available reagents and instruments in laboratories that regularly participate and pass at internal and external quality assessments and proficiency testing.

### Statistical analysis

Results of clinical data and laboratory measurements are presented as mean ± standard deviation (SD) for normally distributed continuous variables or as median and interquartile range [IQR] for non-normally distributed continuous variables. Categorical data are expressed as percentages. Differences between the low-risk and high-risk clusters were compared using the nonparametric Mann–Whitney U test for continuous data and the chi-squared test for categorical data. To compare survival between clusters, hazard ratios (HRs) and 95% confidence intervals (95%CIs) were calculated using the Cox proportional hazards regression model. Non-normally distributed data were log-transformed before analysis. Time to death was defined as time to death or time from study enrollment to last follow-up for censored individuals. Quantile ranges were used to identify outliers in coagulation variables, defined as values outside the 3*(90th − 10th percentile) range of the respective coagulation parameter. Therefore, low and high thresholds were calculated and values outside these thresholds were defined as outliers. Individuals with outliers in coagulation parameters were excluded from the final analysis.

**Fig. 2:**
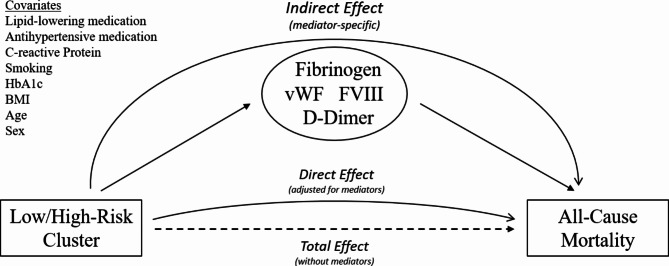
Mediation analysis to evaluate the association of hemostatic factors with cluster-specific risk of death

Mediation analysis was utilized to compute the indirect effects of cluster membership on survival through the mediator candidates, specifically hemostatic factors. Figure 2 schematically illustrates the mediation analysis for the current study: cluster membership and all-cause mortality are the independent and dependent variables, respectively. The total effect represents the relationship between the cluster membership and all-cause mortality. The direct effect additionally adjusts for the mediator candidates. Accordingly, the indirect effect is the extent to which cluster membership has an effect on all-cause mortality through the mediator. For this approach, the effects of cluster membership on each mediator candidate were initially estimated using a simple regression model that predicted the respective variables individually using the cluster membership variable. The regression coefficients obtained from these estimations were multiplied by the coefficients of the respective variables in a Cox regression model that predicted hazard ratios using all mediator candidates separately or simultaneously. The resulting products indicate the extent to which cluster membership affects survival through specific mediator candidates, while controlling for direct and indirect effects via other mediators. Bootstrapped confidence intervals (95% two-tailed) were obtained based on 10,000 resamples to test the resulting indirect effects. The significance of individual indirect effects is determined by whether their confidence intervals include the value of zero. If zero is not within the confidence interval, the indirect effect is considered different from zero. If zero is included in the confidence interval, it is plausible that the indirect effect could be zero in the respective population. Therefore, in these cases, the null hypothesis is retained. To calculate the composite variable vWF:FVIII, the geometric mean of the logarithmic values of vWF and FVIII were calculated for each participant. The resulting vWF:FVIII values were then re-exponentiated and used for the mediation analysis.

A p-value < 0.05 was considered statistically significant. The statistical analysis was performed using GraphPad Prism Software (version 10.1.1; GraphPad Software Inc., San Diego, United States) and R (version 4.3.2; R Core Team, R Foundation for Statistical Computing, Vienna, Austria). Figures were created using GraphPad Prism Software or Microsoft PowerPoint 2019 (Microsoft Corporation, Redmond, United States).

## Results

The analysis included 917 individuals who were assigned to one of the six prediabetes clusters, 643 to the low-risk group, which comprised clusters 1 (*n* = 277), 2 (*n* = 175), and 4 (*n* = 191), and 274 individuals to the high-risk group, including clusters 3 (*n* = 83), 5 (*n* = 78), and 6 (*n* = 113), indicating an increased risk for developing diabetes mellitus and metabolic complications. Table 1 shows the clinical and laboratory characteristics of both groups.

**Table 1:** Clinical and laboratory characteristics of prediabetes clusters

Clusters	Low-Risk	High-Risk	*p*-value
**N**	643	274	
**Sex (female/male**,** %)**	184/459 (28.5/71.5)	74/200 (27.0/73.0)	0.6314
**Age (years)**	60.6 [53.3–67.7]	61.2 [54.8–68.4]	0.3333
**Body mass index (kg/m**^**2**^**)**	25.8 [24.1–27.9]	28.8 [26.6–31.5]	**< 0.0001**
**Glycosylated hemoglobin**,** HbA1c (%)**	5.7 [5.4–6.0]	5.8 [5.5–6.1]	**0.0062**
**Fasting glucose (mg/dL)**	85 [79–91]	93 [85–100]	**< 0.0001**
**Triglycerides (mg/dL)**	122 [94–167]	164 [119–224]	**< 0.0001**
**C-reactive protein (mg/dL)**^**A**^	0.22 [0.01–0.52]	0.35 [0.13–0.73]	**< 0.0001**
**Hypertension**	570 (88.6)	263 (96.0)	**0.0002**
**Antihypertensive medication**	511 (79.4)	233 (85.0)	0.0528
**Cholesterol-lowering medication**	275 (42.8)	126 (46.0)	0.3833
**Cotinine > 15 µg/L**	113 (17.6)	44 (16.1)	0.6324
**All-cause death (%)**	216 (33.6)	112 (40.9)	**0.0420**
**LDL cholesterol (mg/dL)**	120 [100–142]	120 [101–140]	0.5388
**HDL cholesterol (mg/dL)**	41 [35–49]	37 [32–44]	**< 0.0001**
**Total protein (g/dL)**	6.8 [6.5–7.1]	7.0 [6.6–7.3]	**0.0006**
**Albumin (g/dL)**^**B**^	4.4 [4.1–4.8]	4.4 [4.1–4.8]	0.9723
**Ferritin (ng/mL)**	133 [78–233]	163 [96–284]	**0.0014**
**Transferrin (mg/dL)**	248 [226–273]	252 [230–279]	**0.0325**
**Iron (µg/dL)**	98 [77–122]	93 [76–113]	0.1104
**Lactate dehydrogenase (U/L)**	157 [139–177]	165 [147–184]	**0.0002**
**Cholinesterase (U/L)**^**A**^	5690 [4950–6530]	5880 [5130–6740]	**0.0149**
**Amylase (U/L)**	19 [15–24]	19 [14–23]	**0.0490**
**Alkaline phosphatase (U/L)**	109 [91–128]	114 [96–131]	**0.0307**
**Gamma glutamyl transferase (U/L)**	13 [9–22]	17 [11–31]	**< 0.0001**
**Total bilirubin (mg/dL)**	0.6 [0.4–0.8]	0.5 [0.4–0.7]	0.0538
**Creatine kinase (U/L)**	31 [23–43]	33 [24–47]	0.1008
**Aldosterone (ng/L)**^**C**^	84 [54–129]	93 [55–144]	0.0773
**Renin (U/L)**^**A**^	16 [9–32]	19 [10–38]	**0.0114**
**Cortisol (mg/L)**	21 [17–26]	21 [17–26]	0.5750
**Folic acid (µg/L)**	7.9 [6.1–10.1]	7.6 [5.8–10.6]	0.4810
**eGFR CKD-EPI**^**D**^	93 [83–106]	86 [74–99]	**< 0.0001**
**eGFR MDRD**^**D**^	85 [76–95]	82 [73–93]	**0.0144**
**Coronary artery disease (CAD)**^**E**^			
**Normal (smooth contours)**	195 (30.8)	56 (20.7)	**0.0055**
**Minor disease (11–49%)**	57 (9.0)	35 (13.0)	
**1 vessel disease (≥ 50%)**	113 (18.0)	49 (18.1)	
**2 vessel disease (≥ 50%)**	111 (17.5)	49 (18.1)	
**3 vessel disease (≥ 50%)**	158 (25.0)	81 (30.0)	
**> 10% max. stenosis**	466 (73.5)	225 (83.3)	**0.0015**
**> 20% max. stenosis**	439 (69.2)	214 (79.2)	**0.0020**
**> 50% max. stenosis**	382 (60.3)	179 (66.2)	0.0994

Data were available from the following number of participants: ^A^916, ^B^763, ^C^893, ^D^915, ^E^904

Bold values indicate statistical significance

### All-cause mortality in prediabetes clusters

During the median follow-up of 16.9 [11.1–18.0] years, 328 deaths from any cause occurred. The Kaplan-Meier estimator showed no significant differences in survival among the six prediabetes clusters (log-rank test *p* = 0.56). In the unadjusted Cox regression model (model 1), the resulting hazard ratios did not differ significantly between the clusters. After adjustment for age, sex, BMI and HbA1c (model 2), cluster 6 showed a significantly increased risk of death compared to cluster 1 (hazard ratio (HR) = 1.53, confidence interval (CI): 1.03 to 2.29; *p* = 0.0370). In model 3, additionally adjusted for smoking, C-reactive protein (CRP), antihypertensive and lipid-lowering medication, clusters 6 (HR = 1.52, CI: 1.01 to 2.28; *p* = 0.0426) and 4 (HR = 1.48, CI: 1.01 to 2.17; *p* = 0.0427) showed significantly increased risks of death compared to cluster 1. When analyzing the pooled subgroups, the high-risk clusters showed a significantly increased risk of death compared to the low-risk clusters (model 3: HR = 1.30, CI: 1.01–1.67; *p* = 0.0384).

### Differences in coagulation patterns between prediabetes clusters

**Fig. 3:**
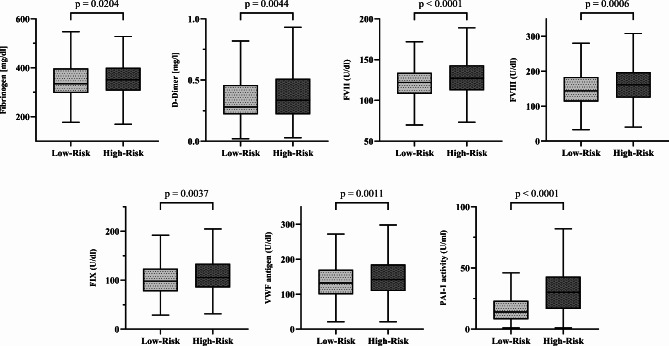
Differences in the coagulation patterns between the low-risk and high-risk prediabetes clusters

We found significant differences in the plasmatic coagulation system between the low-risk and high-risk clusters (see Table 2; Fig. 3; Supplementary Table S2 shows all six clusters). In unadjusted models, individuals in the high-risk clusters had significantly higher concentrations of fibrinogen (+ 5.1%) and D-dimers (+ 21.4%) compared to the low-risk clusters. Additionally, the high-risk clusters showed significantly increased activities of the procoagulant factors FVII (+ 4.1%), FVIII (+ 11.8%), and FIX (+ 10.1%), of von-Willebrand factor (vWF, + 7.6%) and of plasminogen activator inhibitor-1 activity (PAI-1, + 114.3%). In models adjusted for confounders, such as age, sex and BMI (see Supplementary Tables S3 and S4) that affect metabolic clustering and coagulation variables, FVII, FVIII, vWF, and PAI-1 remained significantly higher in the high-risk compared to the low-risk clusters. No significant differences were found between the two groups for the other parameters evaluated.

**Table 2:** Coagulation patterns of prediabetes clusters

	Low-Risk (*N* = 643)	High-Risk (*N* = 274)	Difference ( %)	*p*-value	*p*-value^A^	*p*-value^B^
**PT (%)**	96 [90–100]	96 [91–100]	0.0	0.4636	0.9488	0.9138
**INR**	1.03 [0.99–1.08]	1.03 [0.98–1.07]	0.0	0.4185	0.7310	0.6853
**aPTT (sec)**	33 [31–35]	32 [31–34]	–3.0	0.2266	0.8606	0.5879
**Fibrinogen (mg/dL)**	335 [297–398]	352 [307–400]	+ 5.1	**0.0204**	0.4576	0.1136
**D-dimer (mg/L)**	0.28 [0.22–0.46]	0.34 [0.22–0.51]	+ 21.4	**0.0044**	**0.0347**	0.1913
**FII (U/mL)**	109 [96–120]	109 [96–121]	0.0	0.6297	0.3833	0.5594
**FVII (U/mL)**	122 [108–134]	127 [112–143]	+ 4.1	**< 0.0001**	**0.0002**	**0.0001**
**FVIII (U/mL)**	144 [113–184]	161 [124–198]	+ 11.8	**0.0006**	**0.0021**	**0.0175**
**FIX (U/mL)**^**A**^	98 [77–124]	105 [85–134]	+ 10.1	**0.0037**	**0.0130**	0.0892
**FXI (U/mL)**^**B**^	111 [89–139]	110 [88–136]	+ 0.0	0.8832	0.7291	0.4798
**VWF antigen (U/mL)**	132 [100–170]	142 [109–185]	+ 7.6	**0.0011**	**0.0040**	**0.0486**
**PAI-1 activity (U/mL)**^**C**^	14 [8–23]	30 [16–43]	+ 114.3	**< 0.0001**	**< 0.0001**	**< 0.0001**

Data were available from the following number of participants: ^A^ 871, ^B^ 878, ^C^ 911

^A^p-value adjusted for age, sex, BMI and HbA1c

^B^p-value adjusted for age, sex, BMI, HbA1c, smoking, CRP, antihypertensive and lipid-lowering medication

### Coagulation and all-cause mortality

Survival analysis was used to assess the association between hemostatic factors and all-cause mortality. Therefore, unadjusted and adjusted proportional hazards models were calculated. The unadjusted continuous model showed that increased levels of fibrinogen, D-dimers, FVIII and vWF were significantly associated with increased risk of death (see model 1 in Table 3). Adjustment for the confounders of age, sex, BMI, and HbA1c (model 2) did not alter the results. After additional adjustment for smoking, CRP, antihypertensive medication, and lipid-lowering medication (model 3), elevated levels of FVIII and vWF remained significantly associated with an increased risk of death.

**Table 3:** Association of coagulation parameters with all-cause mortality in the entire cohort

	Model 1	Model 2	Model 3
**Fibrinogen (mg/dL)**	3.682 (2.357–5.753)*	2.278 (1.444–3.595)*	1.761 (0.982–3.155)
**D-dimer (mg/L)**	1.579 (1.365–1.827)*	1.175 (1.011–1.369)*	1.111 (0.953–1.294)
**FII (U/L)**	0.762 (0.538–1.158)	1.212 (0.733–2.004)	1.258 (0.815–2.149)
**FVII (U/L)**	0.745 (0.482–1.204)	0.731 (0.447–1.253)	0.692 (0.422–1.184)
**FVIII (U/L)**	2.456 (1.799–3.356)*	1.515 (1.097–2.091)*	1.430 (1.027–2.001)*
**FIX (U/L)**	1.049 (0.768–1.431)	1.009 (0.731–1.390)	0.952 (0.680–1.331)
**FXI (U/L)**	0.890 (0.649–1.226)	1.079 (0.771–1.512)	1.044 (0.744–1.466)
**VWF antigen (U/L)**	2.977 (2.222–3.993)*	1.682 (1.253–2.259)*	1.538 (1.133–2.089)*
**PAI-1 activity (U/L)**	0.968 (0.860–1.089)	1.084 (0.947–1.244)	1.038 (0.904–1.194)

Shown are hazard ratios and confidence intervals obtained from proportional hazards models

Bold values and asterisks indicate statistical significance

Model 1: unadjusted

Model 2: adjusted for age, sex, BMI and HbA1c

Model 3: adjusted for age, sex, BMI, HbA1c, smoking, CRP, antihypertensive and lipid-lowering medication

### Impact of cluster-specific coagulation patterns on all-cause mortality

Mediation analyses were performed to assess possible causal effects of cluster-specific coagulation patterns on all-cause mortality. Therefore, the indirect (mediated) effects of cluster membership on mortality through hemostatic factors were calculated. We therefore employed the hemostatic factors that were significantly associated with increased all-cause mortality in the entire cohort. First, univariate mediation models were examined. They revealed that cluster-membership related all-cause mortality is significantly mediated by vWF (β = 0.027 95% Bootstrap CI: 0.002 to 0.068, see Table 4). To account for the interrelationship of coagulation proteins, we next performed multiple mediation models. Since vWF is the carrier protein of FVIII in vivo and both parameters were therefore highly correlated (Spearman’s ρ = 0.72, *p* < 0.0001), three different multiple models were run. The initial multiple mediation model, which did not include vWF, indicated that none of the investigated hemostatic factors had a significant mediation effect. The second model, which excluded FVIII, demonstrated that vWF had a significant indirect effect on cluster-related all-cause mortality (β = 0.024 95% Bootstrap CI: 0.001 to 0.065). In the third model, we calculated the new variable vWF:FVIII (see statistical analysis) to assess possible joint or opposing effects of vWF and FVIII. The results of this multiple mediation analysis, including the new variable, revealed a significant positive mediation effect of the composite variable vWF:FVIII on cluster-related all-cause mortality (β = 0.027 95% Bootstrap CI: 0.001 to 0.066).

**Table 4:** Results of mediation analysis of hemostatic factors on cluster-related risk for all-cause mortality

	Univariate Mediation	Multiple Mediation		
**Fibrinogen (mg/dL)**	– 0.006 (**– **0.030–0.013)	– 0.004 (– 0.025–0.010)	– 0.004 (– 0.023–0.009)	– 0.003 (– 0.023–0.009)
**D-dimer (mg/L)**	0.010 (– 0.005–0.037)	0.005 (– 0.012–0.030)	0.005 (– 0.012–0.028)	0.005 (– 0.013–0.028)
**FVIII (U/L)**	0.019 (– 0.003–0.050)	0.015 (– 0.007–0.045)	**-**	**vWF:FVIII** 0.027 (0.001–0.066) *
**vWF antigen (U/L)**	0.027 (0.002–0.068) *	-	0.024 (0.001–0.065) *	

Shown are estimates and 95% bootstrap confidence intervals of indirect effects of cluster membership on survival via hemostatic factors in mediation analysis. Mediation models were adjusted for age, sex, BMI, HbA1c, smoking, CRP, antihypertensive and lipid-lowering medication. Bold values and asterisks indicate statistical significance

## Discussion

Our study examined differences in plasmatic coagulation between prediabetes clusters and assessed if alterations in hemostasis could contribute to their differences in risk for all-cause mortality [6].

We discovered that the prediabetes clusters are linked to specific changes in the plasmatic coagulation system, indicating a prothrombotic and hypofibrinolytic state in the high-risk clusters compared to the low-risk clusters. This suggests a clinically relevant hypercoagulability that increases the risk for atherothrombosis in the high-risk clusters. We also found that elevated levels of fibrinogen, D-dimers, FVIII and von-Willebrand factor (vWF) were associated with an increased risk of mortality, indicating that plasmatic coagulation plays a critical role in stratifying survival among our individuals at cardiometabolic risk. Of note, our mediation analysis indicates that vWF, which is a marker of endothelial dysfunction, significantly contributes to the risk of mortality in the prediabetes high-risk cluster. This finding highlights the importance of endothelial dysfunction as underlying mechanism for the association between metabolic risk, cardiovascular disease and survival.

Prothrombotic factors, specifically fibrinogen, FVII, and FVIII, were markedly elevated in patients of the high-risk clusters. Even after the appropriate adjustments for important confounders, FVII and FVIII were significantly elevated, while the differences in fibrinogen levels disappeared. FVII and FVIII are well established to cardiovascular and ischemic diseases [22–24]. Several studies have shown that high levels of FVIII predict arterial and venous thrombosis. Hence, our current findings indicate that individuals in the high-risk clusters are prone to thrombotic and thromboembolic diseases [25].

Studies have shown that an increase in fasting plasma glucose is associated with an increase in FVIII, regardless of sex, age, and BMI [13]. Our findings show that FVIII levels are significantly higher in the high-risk clusters, independent of age, sex, BMI and also HbA1c. Therefore, this effect is likely not just a consequence of altered glucose metabolism or obesity in the high-risk cluster. Additionally, the differences between high-risk and low-risk clusters were independent of traditional cardiovascular risk factors, such as hypertension, dyslipidemia, inflammation and smoking.

Regarding the association between the prothrombotic coagulation proteins and all-cause mortality, we found that only elevated levels of FVIII, but not FVII, are associated with an increased risk of death. This is consistent with previous results from larger studies that have shown that FVIII, but not FVII, is associated with a higher risk of cardiovascular events and mortality [17, 20, 26]. Our current findings extend this by indicating that FVIII is an independent predictor of premature mortality in prediabetes clusters.

Similar to these prothrombotic factors, vWF was elevated in patients of our high-risk clusters. VWF is the carrier protein of FVIII and plays a pivotal role in thromboembolic cardiovascular events. It may be a predictor of adverse clinical outcome in patients with acute coronary syndrome [27]. Particularly in type 2 diabetes and prediabetes, vWF plays a dominant role in the development of cardiovascular disease and is closely associated with increased CVD risk and all-cause mortality in this population [1, 15]. In our study, we found that both FVIII and vWF were predictors of all-cause mortality, independent of relevant confounders. This is consistent with the results of a previous study that showed that high FVIII and vWF levels were significantly associated with an increased risk of death in patients with venous thrombosis and also in individuals from the general population [20]. However, the authors reported that the relationship between FVIII and vWF levels and risk of death was influenced by confounding factors such as comorbidities and inflammation. Therefore, they concluded that FVIII and vWF are likely not causally related to the risk of death. In our study, we identified FVIII and vWF levels as predictors of all-cause mortality, independently of inflammation and traditional cardiovascular risk factors. Hence, inflammation is likely not the underlying link between FVIII and vWF and mortality in our patients, suggesting a potential causal direct contribution of coagulation. This interpretation is consistent with results of a Mendelian randomization analysis that also identified FVIII and vWF as causally related to cardiovascular events such as CAD, venous thrombosis, and ischemic stroke [28]. In our study, we used univariate and multiple mediation analysis to explore the potential mediating effect of hemostatic factors on increased risk of death in our high-risk clusters. Univariate analysis showed that vWF mediated the cluster-specific mortality risk. However, this model does not take into account the complex interaction between coagulation proteins and can therefore only be considered as indicative. To address the mediation effect of vWF and FVIII separately, but in relation to the other evaluated hemostatic factors, we ran two mediation models: in the first multiple model, FVIII was ignored, and in the second one vWF was ignored. In a further model, taking into account potential synergistic effects of both parameters, we ran a model using the newly generated combined variable vWF: FVIII. Overall, the results of the mediation models indicate that vWF has a significant mediating effect on the cluster-specific risk of death. This effect is independent of the other included hemostatic factors and relevant confounders. This argues against a major confounding of vWF by inflammation and obesity and suggests that vWF alone is the major contributor of the coagulation system to cluster-specific mortality risk. In addition, a small part of the mediation effect can be explained by the interaction between vWF and FVIII, consistent with their known clinically relevant relationship [29, 30]. Indeed, studies on their interplay suggest that vWF is a biomarker of cardiovascular disease rather than a classical procoagulant agent, while FVIII levels have been associated with short-term effects such as hyperglycemia and dyslipidemia, independent of vWF, suggesting a predominant procoagulant function of FVIII [31–33]. Besides this, vWF is widely recognized as a marker of endothelial dysfunction, an important pathophysiological process underlying cardiovascular disease in individuals with prediabetes and diabetes [34, 35]. Therefore, endothelial dysfunction may explain the association between glycemic disorders, coagulation alterations and cardiovascular disease, as shown in our study. Consequently, our current findings indicate that individuals in the high-risk clusters are at greater risk for premature death due to advanced endothelial dysfunction, as indicated by high vWF levels in this subgroup. In line, individuals in the high-risk clusters are known to have higher intima-media thickness [5], a marker of endothelial damage [36], compared to those in the low-risk clusters.

In addition, several mechanisms have been proposed for the association between blood clotting abnormalities and prediabetes [37]. High glucose concentrations induce oxidative stress in vascular endothelial cells which stimulate the release of tissue factor and procoagulant proteins and could thereby promote coagulation [38]. Hyperglycemia may also have a direct stimulatory effect on PAI-1 gene transcription, promoting a hypofibrinolytic state [39]. Moreover, insulin resistance is associated with platelet hypersensitivity which can be explained by an impaired insulin signaling in platelets caused by endothelial dysfunction, hyperglycemia, and oxidative stress [40]. Obesity, a common comorbidity of prediabetes, is also associated with prothrombotic coagulation alterations [9]. This may be explained by increased secretion of PAI-1 from adipocytes and secondary effects of adipokines and free fatty acids that stimulate hepatic synthesis of coagulation proteins and affect the endothelium and platelet activity [9]. Consequently, these mechanisms promote a prothrombotic state, that may link prediabetes and coagulation abnormalities to an increased risk of cardiovascular disease and premature death.

Among the evaluated hemostatic factors, PAI-1 exhibited largest difference between patients in the high-risk and the low-risk clusters. Unlike prothrombotic factors, PAI-1 inhibits fibrinolysis, thereby increasing the risk of thrombotic diseases and events [41]. Several studies have demonstrated that PAI-1 is linked to an increased risk of cardiovascular events [14, 42, 43]. Additionally, PAI-1 has been reported to predict mortality in certain diseases, such as acute myocardial infarction or sepsis [44, 45]. However, our analysis did not find any association between PAI-1 levels and the risk of death in the investigated cohort. The differences in PAI-1 between the low-risk and high-risk cluster may rather reflect their metabolic features than differences in fibrinolysis [46]. Notably, PAI-1 was elevated in the high-risk group, regardless of metabolic factors such as BMI or HbA1c. This supports the finding that PAI-1 may be an independent predictor for the risk of progression to type 2 diabetes and cardiometabolic complications, in parallel with its function as a fibrinolysis inhibitor [47]. Additionally, PAI-1, like vWF, is an established marker of endothelial dysfunction, confirming the association between glycemic disorders, endothelial dysfunction, and cardiovascular disease. [34, 35].

Regarding the potential underlying mechanisms of the cluster-specific coagulation patterns, metabolic variables that were also used for the clustering approach may play a central role. Although the differences in hemostatic factors were independent of age, sex, BMI and HbA1c, hemostatic alterations are likely affected by body fat distribution and glucose metabolism [8]. In a previous study, we showed that fat accumulation in various body parts is linked to increased levels of prothrombotic proteins, such as fibrinogen, D-dimer, FVII, and FVIII. Additionally, especially liver fat content was highly correlated with fibrinogen, FVII, and FVIII levels. This is consistent with both the present findings and the original study on prediabetes clustering, which identified elevated liver fat content in high-risk clusters, specifically clusters 5 and 6 [5]. Furthermore, studies showed that non-alcoholic fatty liver disease is associated with hemostatic alterations that are known to increase the risk of cardiovascular disease [48, 49]. Therefore, we hypothesize that the accumulation of fat in the liver contributes to the hypercoagulable state that we detected in high-risk clusters. Vice versa, personalized prevention and treatment methods, such as lifestyle intervention programs demonstrated that weight loss, but particularly liver fat reduction, is able to revert the prothrombotic potential in individuals with prediabetes and diabetes [50]. Specifically, vWF and PAI-1 levels decreased after the 1-year lifestyle intervention in individuals with prediabetes, suggesting improved endothelial function. However, these results were based on a short follow-up period. Therefore, further longitudinal studies are needed to evaluate the impact of lifestyle intervention programs on the coagulation system and to specifically address the role of vWF on cluster-specific risk of premature death. Because the reduction in vWF during lifestyle intervention was associated with a reduction in liver fat content and systemic subclinical inflammation, therapeutic strategies targeting liver fat and subclinical inflammation may be a promising approach. Of note, longitudinal intervention studies that include precise metabolic phenotyping of participants may also be helpful in delineating underlying mechanistic relationships between coagulation and metabolic variables and cardiovascular endpoints. In addition, future studies could include investigations of platelet function and extracellular vesicles, which are also important regulators of the coagulation system [9, 51, 52].

Clinical implications of our study include vWF as a potential therapeutic target to prevent premature death in persons assigned to the high-risk clusters. Furthermore, regular monitoring of individuals in the high-risk clusters for thrombotic diseases may be useful, as our findings suggest a hypercoagulable state in these individuals. In addition, measurements of endothelial function (circulating markers and ultrasound) may help to further assess the risk of atherosclerosis and cardiovascular disease. In general, endothelial dysfunction is a reversible disease and there are several treatment options to improve endothelial function. Therefore, risk stratification and regular monitoring of endothelial function should be considered in individuals with prediabetes.

In conclusion, our current findings highlight the central role of metabolic subphenotypes in the stratification of individuals at cardiometabolic risk. Prediabetes high-risk clusters were associated with a clinically significant hypercoagulable state. Importantly, hemostatic factors likely play a critical role in cluster-specific mortality risk. Therefore, in addition to the established differences in risk for type 2 diabetes, prediabetes clusters can stratify individuals according to their risk for cardiovascular and thrombotic disease and premature death. Hence, prediabetes high-risk clusters are characterized by prothrombotic changes that predisposes them for premature death. This underscores the need for considering hypercoagulable states in metabolic risk stratification and treatment strategies.

## Electronic supplementary material

Supplementary Material 1.

## Data Availability

The data are not publicly available because they contain information that could compromise the privacy/consent of research participants.

## Abbreviations

PT: Prothrombin time
INR: International normalized ratio
aPTT: Activated partial thromboplastin time
F: Factor
VWF: Von-willebrand factor
PAI-1: Plasminogen activator inhibitor-1
