# Supplementary material for: Blood coagulation in Prediabetes clusters–impact on all-cause mortality in individuals undergoing coronary angiography
Source: Cardiovasc Diabetol. 2024 Aug 22;23:306. doi: 10.1186/s12933-024-02402-z (PMC11342575; doi:10.1186/s12933-024-02402-z)
Supplement: Supplementary file 1 — Supplementary Material 1. [file 12933_2024_2402_MOESM1_ESM.docx]

**SUPPLEMENTARY DATA**

**Supplementary Table S1: Outliers in coagulation parameters.** Shown are the number of outliers and the raw values of each coagulation parameter.

| **Coagulation parameters** | **Outliers** |
| --- | --- |
| **PT (%)** | 2 (33%/33%) |
| **INR** | 4 (1.83/2.22/2.64/2.65) |
| **aPTT (sec)** | 11 (66 sec /72 sec /73 sec /73 sec /74 sec /82 sec /91 sec /94 sec /95 sec /104 sec /125 sec) |
| **Fibrinogen (mg/dl)** | 0 |
| **D-dimer (mg/L)** | 12 (3.3 mg/L/3.4 mg/L /3.5 mg/L /3.6 mg/L /3.8 mg/L /3.9 mg/L /5.1 mg/L /5.2 mg/L /5.3 mg/L /7.1 mg/L /16.1 mg/L /22 mg/L) |
| **FII (U/mL)** | 0 |
| **FVII (U/mL)** | 0 |
| **FVIII (U/mL)** | 0 |
| **FIX (U/mL) ^A^** | 0 |
| **FXI (U/mL) ^B^** | 0 |
| **VWF antigen (U/mL)** | 0 |
| **PAI-1 activity (U/mL) ^C^** | 1 (172 U/mL) |

Abbreviations: PT, prothrombin time; INR, international normalized ratio; aPTT, activated partial thromboplastin time; F, factor; VWF, von-willebrand factor; PAI-1, plasminogen activator inhibitor-1.

**Supplementary Table S2: Coagulation patterns in prediabetes clusters.**

| **Cluster** | **1** | **2** | **3** | **4** | **5** | **6** |
| --- | --- | --- | --- | --- | --- | --- |
| **High/Low-Risk Cluster** | Low | Low | High | Low | High | High |
| **N** | 277 | 175 | 83 | 191 | 78 | 113 |
| **PT (%)** | 97 [90 – 100] | 96 [90 – 100] | 99 [91- 100] | 96 [91- 100] | 99 [92- 100] | 96 [90 – 100] |
| **INR** | 1.02 [0.98 – 1.08 | 1.04 [0.99 – 1.08 | 1.01 [0.98 – 1.07 | 1.03 [0.99 – 1.08 | 1.03 [0.99 – 1.06 | 1.03 [0.99 – 1.08 |
| **aPTT (sec)** | 33 [31 – 36] | 33 [31 – 35] | 32 [30 – 34] | 33 [31 – 34] | 32 [31 – 34] | 33 [31 – 35] |
| **Fibrinogen (mg/dl)** | 346 [300 – 414] | 321 [277 – 363] | 348 [301 – 394] | 348 [309 – 408] | 363 [316 – 413] | 352 [309 – 391] |
| **D-dimer (mg/L)** | 0.30 [0.22 – 0.50] | 0.26 [0.22 – 0.40] | 0.33 [0.22 – 0.47] | 0.27 [0.22 – 0.49] | 0.35 [0.23 – 0.61] | 0.34 [0.22 – 0.52] |
| **FII (U/mL)** | 110 [98 – 121] | 103 [93 – 117] | 109 [98 – 122] | 112 [97 – 120] | 111 [98 – 121] | 107 [94 – 120] |
| **FVII (U/mL)** | 124 [109 – 134] | 118 [104 – 128] | 126 [111 – 143] | 125 [110 – 137] | 133 [114 – 151] | 126 [111 – 142] |
| **FVIII (U/mL)** | 148 [114 – 191] | 142 [112 – 176] | 156 [126 – 196] | 138[112 – 184] | 149 [115 – 195] | 166 [133 – 198] |
| **FIX (U/mL) ^A^** | 101 [78 – 130] | 93 [76 – 119] | 101 [86 – 127] | 97 [79 – 124] | 112 [84 – 143] | 105 [85 – 126] |
| **FXI (U/mL) ^B^** | 110 [89 – 139] | 112 [89 – 139] | 113 [89 – 139] | 111 [89 – 138] | 112 [88 – 138] | 107 [89 – 135] |
| **VWF antigen (U/mL)** | 132 [100 – 172] | 130 [98 – 164] | 142 [103 – 174] | 132 [103 – 162] | 128 [102 – 193] | 150 [122 – 190] |
| **PAI-1 activity (U/mL) ^C^** | 14 [8 – 24] | 11 [6 – 18] | 23 [14 – 34] | 17 [10 – 29] | 38 [23 – 53] | 32 [19 – 47] |

Data were available from the following number of participants: ^A^ 871, ^B^ 878, ^C^ 911.

Data are presented as median and interquartile range (1^st^ – 3^rd^).

Abbreviations: PT, prothrombin time; aPTT, activated partial thromboplastin time; F, factor; VWF, von-willebrand factor; PAI-1, plasminogen activator inhibitor-1.

**Supplementary Table S3: Correlation of coagulation variables with age and BMI.**

|  | **Age** | **BMI** |
| --- | --- | --- |
| **PT (%)** | -0.08* | -0.29*** |
| **INR** | 0.09** | 0.30*** |
| **aPTT (sec)** | -0.04 | -0.08* |
| **Fibrinogen (mg/dl)** | 0.17*** | 0.10** |
| **D-dimer (mg/L)** | 0.39*** | -0.02 |
| **FII (U/mL)** | -0.12** | 0.05 |
| **FVII (U/mL)** | 0.09** | 0.07* |
| **FVIII (U/mL)** | 0.27*** | 0.04 |
| **FIX (U/mL) ^A^** | 0.03 | 0.06 |
| **FXI (U/mL) ^B^** | -0.04 | -0.01 |
| **VWF antigen (U/mL)** | 0.30*** | 0.03 |
| **PAI-1 activity (U/mL) ^C^** | -0.10** | 0.36*** |

Data were available from the following number of participants: ^A^ 871, ^B^ 878, ^C^ 911.

Shown are Spearman's rank correlation coefficient for each correlation; Asterisks indicate statistical significance: *p≤0.05, **p≤0.01, ***p≤0.001.

Abbreviations: PT, prothrombin time; aPTT, activated partial thromboplastin time; F, factor; VWF, von-willebrand factor; PAI-1, plasminogen activator inhibitor-1.

**Supplementary Table S4: Sex-dependent differences in coagulation variables.**

|  | **Men (N = 659)** | **Women (N = 258)** | **p-value** |
| --- | --- | --- | --- |
| **PT (%)** | 96 [90 – 100] | 99 [94 – 100] | <0.0001 |
| **INR** | 1.03 [0.00 – 1.08] | 1.01 [0.98 – 1.05] | <0.0001 |
| **aPTT (sec)** | 33 [31 – 35] | 32 [30 – 34] | <0.0001 |
| **Fibrinogen (mg/dl)** | 342 [301 – 403] | 344 [300 – 391] | 0.6772 |
| **D-dimer (mg/L)** | 0.28 [0.22 – 0.47] | 0.33 [0.22 – 0.50] | 0.0344 |
| **FII (U/mL)** | 107 [95 – 119] | 116 [98 – 125] | <0.0001 |
| **FVII (U/mL)** | 120 [107 – 132] | 134 [119 – 146] | <0.0001 |
| **FVIII (U/mL)** | 146 [113 – 182] | 157 [118 – 200] | 0.0075 |
| **FIX (U/mL) ^A^** | 101 [80 – 127] | 99 [79 – 128] | 0.7893 |
| **FXI (U/mL) ^B^** | 109 [88 – 135] | 117 [93 – 146] | 0.0017 |
| **VWF antigen (U/mL)** | 134 [102 – 174] | 136 [102 – 174] | 0.7509 |
| **PAI-1 activity (U/mL) ^C^** | 17 [9 – 34] | 17 [9 – 29] | 0.4928 |

Data were available from the following number of participants: ^A^ 871, ^B^ 878, ^C^ 911.

Data are presented as median and interquartile range (1st – 3rd).

Results were compared using the paired Wilcoxon-signed rank test.

Abbreviations: PT, prothrombin time; aPTT, activated partial thromboplastin time; F, factor; VWF, von-willebrand factor; PAI-1, plasminogen activator inhibitor-1.
